# Applying Natural Language Processing to Textual Data From Clinical Data Warehouses: Systematic Review

**DOI:** 10.2196/42477

**Published:** 2023-12-15

**Authors:** Adrien Bazoge, Emmanuel Morin, Béatrice Daille, Pierre-Antoine Gourraud

**Affiliations:** 1 Nantes Université, École Centrale Nantes, CNRS, LS2N, UMR 6004 F-44000 Nantes France; 2 Nantes Université, CHU de Nantes, Pôle Hospitalo-Universitaire 11: Santé Publique, Clinique des données, INSERM, CIC 1413 F-44000 Nantes France; 3 Nantes Université, INSERM, CHU de Nantes, École Centrale Nantes, Centre de Recherche Translationnelle en Transplantation et Immunologie, CR2TI F-44000 Nantes France

**Keywords:** natural language processing, data warehousing, clinical data warehouse, artificial intelligence, AI

## Abstract

**Background:**

In recent years, health data collected during the clinical care process have been often repurposed for secondary use through clinical data warehouses (CDWs), which interconnect disparate data from different sources. A large amount of information of high clinical value is stored in unstructured text format. Natural language processing (NLP), which implements algorithms that can operate on massive unstructured textual data, has the potential to structure the data and make clinical information more accessible.

**Objective:**

The aim of this review was to provide an overview of studies applying NLP to textual data from CDWs. It focuses on identifying the (1) NLP tasks applied to data from CDWs and (2) NLP methods used to tackle these tasks.

**Methods:**

This review was performed according to the PRISMA (Preferred Reporting Items for Systematic Reviews and Meta-Analyses) guidelines. We searched for relevant articles in 3 bibliographic databases: PubMed, Google Scholar, and ACL Anthology. We reviewed the titles and abstracts and included articles according to the following inclusion criteria: (1) focus on NLP applied to textual data from CDWs, (2) articles published between 1995 and 2021, and (3) written in English.

**Results:**

We identified 1353 articles, of which 194 (14.34%) met the inclusion criteria. Among all identified NLP tasks in the included papers, information extraction from clinical text (112/194, 57.7%) and the identification of patients (51/194, 26.3%) were the most frequent tasks. To address the various tasks, symbolic methods were the most common NLP methods (124/232, 53.4%), showing that some tasks can be partially achieved with classical NLP techniques, such as regular expressions or pattern matching that exploit specialized lexica, such as drug lists and terminologies. Machine learning (70/232, 30.2%) and deep learning (38/232, 16.4%) have been increasingly used in recent years, including the most recent approaches based on transformers. NLP methods were mostly applied to English language data (153/194, 78.9%).

**Conclusions:**

CDWs are central to the secondary use of clinical texts for research purposes. Although the use of NLP on data from CDWs is growing, there remain challenges in this field, especially with regard to languages other than English. Clinical NLP is an effective strategy for accessing, extracting, and transforming data from CDWs. Information retrieved with NLP can assist in clinical research and have an impact on clinical practice.

## Introduction

### Background

For >20 years, health data from patient care have been systematically archived in the form of electronic health records (EHRs) [1,2]. Databases have been created to gather both structured data (eg, vital signs and clinical-biological characteristics and demographics) and unstructured data (eg, textual reports of hospitalizations or visits). These large amounts of data involve multiple contributors: patients, for whom data are collected during hospitalizations or visits; caregivers, who care for the patients and collect the data; and health care institutions, which organize all operational and financial logistics involving the care and related data [3]. The first purpose of collecting these data is to broadly deliver high-quality care to patients, even if the data may be repurposed for secondary use, such as reduction in health care costs, population health management, and clinical research [1]. Human data in clinical research are intended for research purposes and limited in terms of sample size, scope, and longitudinal follow-up (ie, clinical trials or disease registries). The secondary use of EHRs allows to increase patient recruitment in trials [4] and enables access to a larger variety of clinical information for clinical research [5,6].

The rapid increase in digital data production prompted the construction of clinical data warehouses (CDWs), also known as health data warehouses or biomedical data warehouses, for the secondary use of EHRs [2]. *CDW* refers to the interconnection of disparate data from different sources, which are restructured into a common format and indexed using standard vocabularies. CDWs collect data from millions of patients treated in hospitals and can be accessed by stakeholders to analyze care situations and make critical decisions [7]. Unlike in the fields of logistics, marketing, and sales, the health care field has been slow to fully integrate data warehouses. CDWs require managing security and privacy constraints related to medical data [7]. Depending on which country houses the CDW, medical data–related policies can vary and potentially slow the construction process [8]. Data warehouses have been part of the health care landscape for decades [9], especially in the United States, where the first CDWs appeared in the 1990s. In some countries, such as France, CDWs have only been constructed more recently owing to policy constraints. At the institutional level, the use of CDWs underscores that organizations recognize the transformative potential and value of the data generated by their activity. This secondary use of data is facilitated by technological advances in artificial intelligence [10]. Among many types of data, textual data reinforce the popularity of a subgroup of artificial intelligence methods, natural language processing (NLP), which implements algorithms that can operate on massive unstructured textual data [11]. The majority of clinical information is stored in unstructured text format, and NLP allows accessing this information [12,13].

### Objectives

This review aims at providing an overview of studies applying clinical NLP to textual data from CDWs. The focus of this review is to identify the (1) NLP tasks applied to data from CDWs and (2) NLP methods used for each task.

## Methods

The PRISMA (Preferred Reporting Items for Systematic Reviews and Meta-Analyses) guidelines were followed for reporting this review (Multimedia Appendix 1).

### Review Method and Selection Criteria

**Figure 1:**
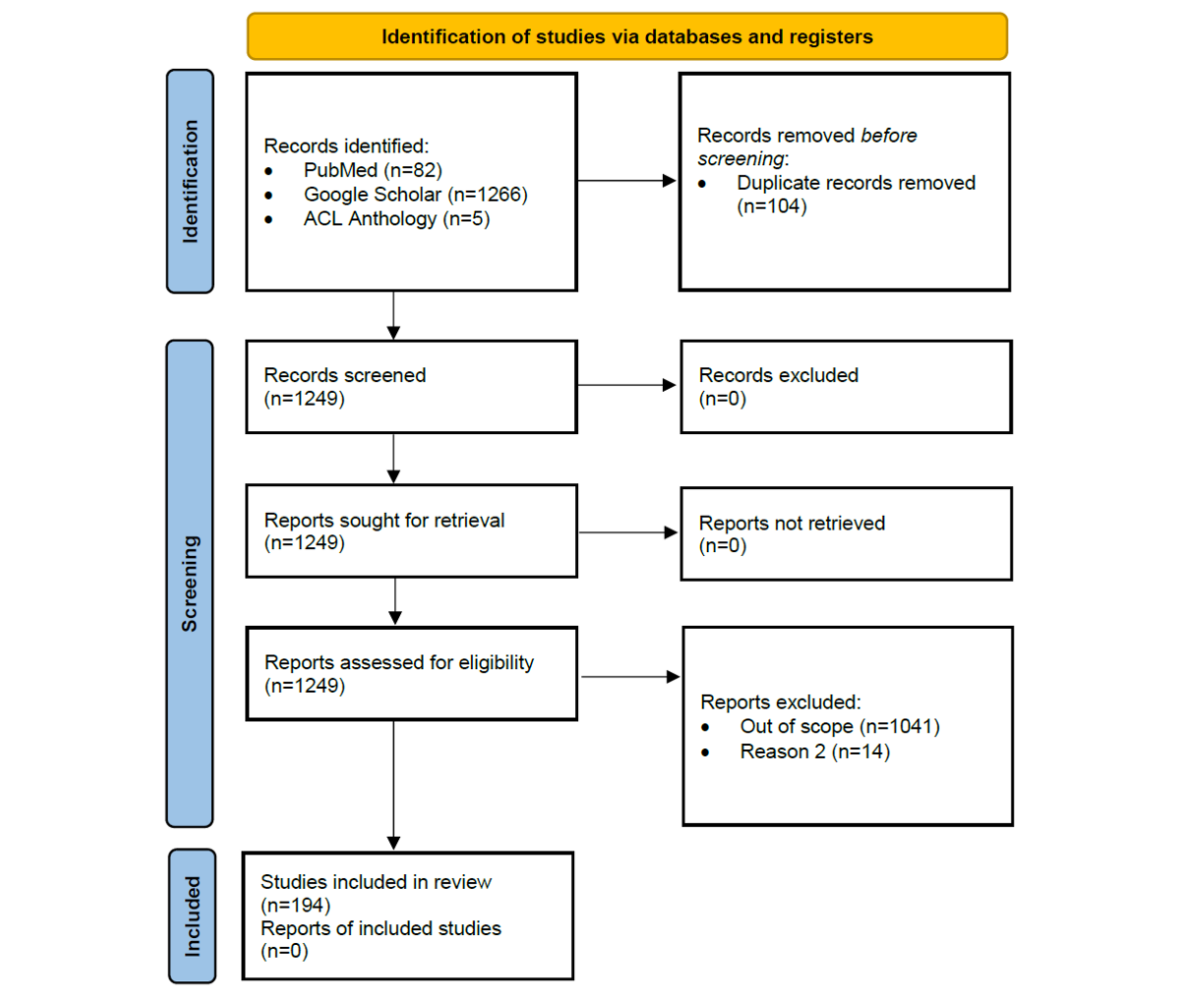
PRISMA (Preferred Reporting Items for Systematic Reviews and Meta-Analyses) article selection flowchart.

Articles identified from the queries were manually included on the basis of the following inclusion criteria: articles (1) mentioning the use of NLP on data from CDWs, (2) published between 1995 and 2021, and (3) written in English. The inclusion was carried out by reading titles and abstracts or by searching the article for the keywords used in the queries to determine whether it was relevant. Details of the article selection steps are described in Figure 1.

### Bibliographic Databases

We searched for relevant articles in 3 bibliographic databases: PubMed, ACL Anthology, and Google Scholar. PubMed is specialized in biomedical literature; its query builder allows searchers to construct queries based on both Medical Subject Headings terms and natural language. ACL Anthology covers the literature published in conferences related to computational linguistics and NLP. Google Scholar does not have a dedicated area of specialty for the papers it references and covers a wide range of the literature.

### Search Strategy

Identifying papers with NLP applied to data from CDWs involved combining multiple designations: the term *data warehouse* is sometimes referred to as a *database* or a *repository*. In addition, the source of the data used in clinical studies may only be listed in the main manuscript. Data collection requires using multiple queries to aim at both high specificity and high sensitivity.

To retrieve a representative selection of papers, we used queries based on specific keywords for each topic of interest, that is, (1) CDWs and (2) NLP:

- CDWs: “clinical data warehouse,” “biomedical data warehouse,” and “health data warehouse.” The selected keywords representing this topic correspond to the most commonly used terms for CDWs.
- NLP: “natural language processing,” “NLP,” and “text mining.” The keyword “text mining” complements the concept of the “natural language processing” keyword. Text mining stands out as the most frequently used NLP application in the medical field. As a result, the term “natural language processing” can sometimes be eclipsed by “text mining.”

Several queries were made using the selected keywords in each bibliographic database. The details of each query are available in Multimedia Appendix 2.

All queries were run on February 23, 2022. PubMed and ACL Anthology papers were retrieved by manually executing queries on the respective websites of these bibliographic databases. Google Scholar papers were collected using free software [14]. The results of the queries were merged, and duplicates were removed.

The queries are not exhaustive but rather aim to provide a limited and representative selection of papers covering the topics of interest. Synonyms for *warehouse*, such as *database* or *repository*, were not used in the queries to avoid the collection of a significant number of irrelevant articles to review. Furthermore, some papers may also apply NLP to data from CDWs without mentioning the CDW and could be missed by the queries.

### Data Collection

The following data were manually collected from the included articles: (1) NLP tasks addressed in the original paper (the NLP task classification is based on the one provided by Névéol et al [13]), (2) NLP methods used to address the tasks, (3) the CDW that is the source of the data, and (4) the language of the data used in the paper.

## Results

### Overview

A total of 1353 articles (PubMed: n=82, 6.06%; Google Scholar: n=1266, 93.57%; and ACL Anthology: n=5, 0.37%) were identified with the initial search strategy. After reviewing the title and abstract of each article, of the 1353 articles, 1159 (85.66%) were excluded owing to duplication (n=104, 8.97%), language issues (n=14, 1.21%), and for being out of the scope of this review (n=1041, 89.82%). Overall, of the initially identified 1353 articles, 194 (14.34%) met the inclusion criteria. These 194 articles were published between 2002 and 2021, which means that articles published between 1995 and 2001 did not meet the inclusion criteria.

This section gathers the topics covered in published research on NLP applied to data from CDWs. The results of the reviewed articles are presented by the NLP task mentioned in the articles. Although many articles address the same NLP task, we decided to not directly compare the performances of the methods used in the articles in this review. Methods have been evaluated with different data in different languages and with different metrics. Hence, we concluded that it was not relevant to perform this comparison.

Table 1 gives the count of studies based on the NLP task for 2 periods of time: 2002-2015 and 2016-2021. The 2 time periods were chosen owing to the transition in the NLP paradigm, shifting from knowledge-based to machine learning methods. This transition coincided with the emergence of new tasks, including language modeling.

**Table 1:** Natural language processing (NLP) tasks reported in the retrieved publications (n=194).

NLP tasks		NLP methods used, n (%)		References
		2002-2015	2016-2021	
**Information extraction (n=112)**				
	Medical concepts (n=37)	S^a^: 14 (74); ML^b^: 5 (26)	S: 10 (40); ML: 11 (44); DL^c^: 4 (16)	[15-51]
	Specific characteristics (n=40)	S: 4 (67); ML: 2 (33)	S: 22 (56); ML: 12 (31); DL: 5 (13)	[52-91]
	Drugs and adverse events (n=26)	S: 10 (77); ML: 3 (23)	S: 8 (57); ML: 1 (7); DL: 5 (36)	[49,52,92-115]
	Findings and symptoms (n=8)	S: 1 (50); ML: 1 (50)	S: 2 (25); ML: 2 (25); DL: 4 (50)	[49,52,116-121]
	Relation extraction (n=1)	S: 1 (100)	N/A^d^	[50]
**Classification (n=51)**				
	Phenotyping (n=38)	S: 7 (78); ML: 2 (22)	S: 17 (49); ML: 12 (34); DL: 6 (17)	[50,122-158]
	Indexing and coding (n=7)	S: 3 (100)	S: 2 (50); ML: 1 (25); DL: 1 (25)	[159-165]
	Topic modeling (n=3)	N/A	S: 1 (25); ML: 3 (75)	[166-168]
	Patient identification (n=3)	N/A	S: 1 (25); ML: 2 (50); DL: 1 (25)	[169-171]
**Context analysis (n=18)**				
	Similarity (n=6)	S: 2 (100)	S: 1 (25); DL: 3 (75)	[172-177]
	Temporality (n=4)	S: 1 (100)	S: 2 (100)	[93,178-180]
	Negation detection (n=3)	N/A	S: 2 (67); DL: 1 (33)	[178,181,182]
	Abbreviation (n=2)	N/A	S: 2 (100)	[183,184]
	Uncertainty (n=1)	N/A	S: 1 (100)	[180]
	Experiencer (n=2)	N/A	S: 2 (100)	[178,182]
Language modeling (n=11)		N/A	ML: 6 (46); DL: 7 (54)	[171,185-194]
**Resource development (n=6)**				
	Corpora and annotation (n=4)	N/A	ML: 1 (100)	[195-198]
	Lexica (n=2)	N/A	S: 2 (67); ML: 1 (33)	[199,200]
Shared tasks (n=5)		S: 4 (57); ML: 3 (43)	S: 1 (100)	[201-205]
Deidentification (n=2)		S: 1 (50); ML: 1 (50)	DL: 1 (100)	[206,207]
Data cleaning (n=1)		N/A	ML: 1 (100)	[208]

^a^S: symbolic methods.

^b^ML: machine learning.

^c^DL: deep learning.

^d^N/A: not applicable.

### Information Extraction

Information extraction is one of the most studied tasks in NLP within the clinical field. In the included articles, named entity recognition (NER) primarily focuses on identifying entities such as protected health information (PHI) to deidentify clinical documents [206,207], as well as various clinical concepts. These concepts encompass diseases [20,25,40,41,45,47,49]; findings and symptoms [49,52,116-119,121]; and medication names [49,52,93-95,99,100,102,106,107,112,113,115], along with their associated details such as dose, frequency, and duration [52,93-95,112,113,115] as well as potential adverse events [96-98,100,101,106-110,114]. These medical concepts can be mapped to terminologies or ontologies such as the Unified Medical Language System (UMLS) [23,24,30,37-39,41,46,97], Systematized Nomenclature of Medicine–Clinical Terms (SNOMED-CT) [27,28,30], or International Classification of Diseases, Ninth Revision (ICD-9) [21].

Several popular NLP systems have been extensively used for extracting, structuring, and encoding clinical information from narrative patient reports in English. Numerous studies detail the application of the Medical Language Extraction and Encoding System (MedLEE) for clinical concepts [24,27-29,32-36,50,51,121] or medication [103,104,111] extraction, as well as UMLS coding. The extraction and mapping of clinical information from clinical notes to UMLS has also been accomplished using the clinical Text Analysis and Knowledge Extraction System (cTAKES) [16,17,20,22,100,129,134,168], MetaMap [31,37,38,47], MedTagger [44,45,67,78,86,105], and the National Center for Biomedical Ontology (NCBO) Annotator [97,99,106,107,109,114]. Extracted concepts can be mapped to other standard ontologies and terminologies, such as SNOMED-CT [27]. Caliskan et al [95] evaluated the Averbis Health Discovery NLP system on a medication extraction task on German clinical notes.

Other systems addressing NER or information extraction were customized to specific use cases. Rule-based methods encoded dictionaries and terminologies to match terms and concepts in clinical texts [40-42,49,102,108,112,113]. Machine learning methods take advantage of the clinical knowledge in the large amount of data in CDWs. According to the time period, methods that were used reflect the trend of using NLP state-of-the-art methods and language models. Conditional random fields (CRFs) were used to extract clinical concepts [23,46] or PHI for the deidentification of clinical documents [207]. Hierarchically supervised latent Dirichlet allocation was applied to hospital discharge summaries to predict ICD-9 codes [21]. Deep learning approaches such as bidirectional long short-term memory–CRF (BiLSTM-CRF) [93,113,115] and recurrent neural network grammars [93] performed medical entity extraction in French clinical texts. Chokshi et al [119] compared a bag-of-words model with support vector machine (SVM) and 2 neural network models: a convolutional neural network (CNN) and a neural attention model, both with Word2Vec embedding as input. The accuracies of the CNN and neural attention model models were relatively equal, but they were higher than the accuracy of the SVM model. Lerner et al [49] compared 3 systems for clinical NER: a terminology-based system built on UMLS and SNOMED-CT, a bidirectional gated recurrent unit–CRF system, and a hybrid system using the prediction of the terminology-based system as a feature for the bidirectional gated recurrent unit–CRF system. Yang et al [206] identified PHI from free text with a long short-term memory (LSTM)–CRF model.

Recent state-of-the-art models based on transformer neural architectures [209] were also applied to extract medical concepts. Neuraz et al [52] used a BiLSTM-CRF layer on top of a vector representation of tokens computed by Bidirectional Encoder Representations from Transformers (BERT) in French. BERT and Robustly Optimized BERT Pretraining Approach were examined to extract social and behavioral determinants of health concepts from clinical narratives [15]. Some of the studies paired a neural language model with simple pattern matching techniques; for example, Jouffroy et al [115] proposed a hybrid approach for the extraction of medication information from French clinical text that combined regular expressions to preannotate the text with contextual word embeddings (embeddings from language models [ELMo]) that are fed into a deep recurrent neural network (BiLSTM-CRF).

Some of the studies (31/194, 16%) addressed specific clinical information extracted from clinical texts. These included bone density [59], breast cancer gene 1 or 2 mentions [86], the predictors and timing of lifestyle modification for patients with hypertension [60], the determination of positivity at imaging presentation in radiology reports [66], Banff classification [69], surgical site infection [70], Breast Imaging Reporting and Database System category 3 [71,72], chemotherapy toxicities [76], vital signs [79], transurethral resection of bladder tumors [80], statin use [57], human leukocyte antigen genotypes [82], unplanned episodes of care [83], smoking status [65,84], monoclonal gammopathy [90], skeletal site-specific fractures [85], and social determinants of health [66]. Methods used to extract this information were rule based [67,69-72,76,79,80,82-85], statistical [59,60], or a combination of both [86,90].

Multiple pieces of information about patients were extracted from clinical texts for application in retrospective studies [56]. Ansoborlo et al [89] extracted 52 pieces of bioclinical information from French multidisciplinary team meeting reports concerning lung cancer by applying regular expressions and then compared this approach with a Bayesian classifier method.

Extracting information from clinical text was also carried out as a prediction task. Predicted data cover length of hospital stay [73], the likelihood of neuroscience intensive care unit admission [64], the risk of 30-day readmission in patients with heart failure [55], or quality metrics for the assessment of pretreatment digital rectal examination documentation [62]. Risk assessments of diseases or pathologies, including HIV [61,81], pancreatic cancer [75], pressure ulcer [91], chronic kidney disease [63], and breast cancer [54], have also been studied as prediction tasks. Predicting this clinical information can be achieved with rule-based methods [73,81], machine learning techniques such as latent Dirichlet allocation [63,73], or a combination of both [75,91].

### Context Analysis

Linguistic occurrences are particularly relevant where medical information is concerned, such as negation, temporality, uncertainty, or experiencer (ie, determine whether the identified information is related to the patient or a third party, such as a family member). In the included studies, rule-based methods were often used to detect contextual information in clinical text [178,180,182]. Although these methods offer good results (with an approximate *F*_1_-measure value of 0.90), they rely on handmade resources, such as terminologies and regular expressions, and customization is often needed for specific use cases. Temporality patterns have been studied by Liu et al [92] to discern adverse drug events from indications in clinical text. Zhou et al [179] describe a temporal constraint structure constructed from temporal expressions in discharge summaries to model these expressions. In the clinical domain, many temporal expressions have unique characteristics, and this structure provides comprehensive coverage in encoding these expressions. Abbreviations are widely used in medicine and have been studied in French [183] and English [184] clinical texts to better handle medical abbreviations. Recent embedding-based methods such as BERT have made it easier to study negation detection [181] and text similarity [173,174]. Text similarity has also been studied to identify semantically similar concepts [175], similar patients [177], or to detect redundancy in clinical texts [172,176].

### Classification

Identifying patients is a key component in clinical research for constructing population studies. NLP can improve the querying and indexing of patients and their data in CDWs. Zhu et al [161] addressed query expansion based on a large in-domain clinical corpus to solve problems of polysemy, synonymy, and hyponymy in clinical text to improve patient identification. Query expansion was also studied through 3 methods: synonym expansion strategy, topic modeling, and a predicate-based strategy derived from MEDLINE abstracts [165]. An automated electronic search algorithm for identifying postoperative complications was evaluated by Tien et al [162]. A semantic health data warehouse was designed to assist health professionals in prescreening eligible patients in clinical trials [163,164]. A combination of structured and unstructured German data was used by Scheurwegs et al [160] to assign clinical codes to patient stays.

Downstream of the query of CDWs, NLP can be applied to identify patients or documents of interest when the classification methods offered by CDWs are not precise enough. Patient identification can be carried out using methods such as rule-based approaches, which involve using terms related to eligible criteria [127,137,140-150,153,170], or learning-based approaches [126,131,133], or a combination of both [152,155-157,169]. Li et al [166] and Chen et al [167] applied latent Dirichlet allocation in clinical notes for topic modeling. Agarwal et al [154] detailed a logistic regression model of phenotypes learned on noisy labeled data. Some of the studies (4/194, 2.1%) relied on Dr Warehouse, a biomedical data warehouse oriented toward clinical narrative reports, developed at Necker Children’s Hospital in Paris, France. This data warehouse was used to explore, using the frequency and term frequency–inverse document frequency (TF-IDF), the association between clinical phenotypes and rare diseases such as the potassium voltage-gated channel subfamily A member 2 variant in neurodevelopmental syndromes [138], Dravet syndrome [125], ciliopathy [139], and other rare diseases [136].

### Language Modeling

Recent word embedding–based methods take advantage of the large amount of data stored in CDWs to learn effective semantic representations of clinical texts. In the included articles, these methods allowed to make calculations on words to find, for example, similar terms in the embedding space [88,130]. Among these methods, transformer-based models, such as BERT, were fine-tuned for multiple tasks, including text classification to map document titles to Logical Observation Identifiers Names and Codes Document Ontology [159] and sequence labeling to detect and estimate the location of abnormalities in whole-body scans [53]. Similarly, clinical text was structured with the classification of ICD-9 codes based on vectorization methods [190,191].

Some of the studies evaluated the effectiveness of word embedding models on multiple tasks. Lee et al [135] evaluated Node2Vec, singular value decomposition, Language Identification for Named Entities, Word2Vec, and global vectors for word representation (GloVe) in retrieving relevant medical features for phenotyping tasks. The authors demonstrated that GloVe, when trained on EHR data, outperforms other embedding methods. GloVe and Word2Vec were used in conjunction with LSTM and gated recurrent unit and evaluated across multiple tasks, with gated recurrent unit outperforming LSTM [192]. Similarly, Dynomant et al [193] compared on multiple tasks 3 embedding methods (Word2Vec, GloVe, and fastText) trained on a French corpus. The 3 methods were evaluated on 4 tasks, and Word2Vec with the skip-gram architecture had the highest score on 3 (75%) of the 4 tasks. Peng et al [185] evaluated 2 transformer-based models, BERT and ELMo, on 10 benchmark data sets and found that the BERT model achieved the best results. BERT was also evaluated on sentence similarity, relation extraction, inference, and NER tasks on data sets from clinical domains [186]. The study by Neuraz et al [188] comparing fastText and ELMo showed that models learned on clinical data performed better than models learned on data from the general domain. The study by Tawfik and Spruit [187] described a toolkit to evaluate the effectiveness of sentence representation learning models.

Text representation models are commonly used as embedding layers in neural network models developed for specific tasks. Word2Vec has been used in numerous studies for various purposes, including assessing bone scan use among patients with prostate cancer with a CNN [151], screening and diagnosing of breast cancer with a deep learning architecture [123], extracting features used for risk prediction of liver transplantation for hepatocellular cancer with a capsule neural network [124], and using a CNN to learn the clinical trial criteria eligibility status of patients for participation in cohort studies [171]. Lee et al [194] proposed a unified graph representation learning framework based on graph convolutional networks and LSTM to construct an EHR graph representation of medical entities. Dligach et al [189] developed a clinical text encoder for specific phenotypes. Experiments were conducted with a deep averaging network and a CNN to construct this text encoder.

### Resource Development and Shared Tasks

Many NLP methods rely on clinically specific resources to be developed. In the included articles, data from CDWs, combined with clinical expert knowledge, allowed the development of resources such as annotation guidelines and schemes [195,196,198], lexica [200], ontologies [199], or frameworks to validate the outputs of NLP systems [197].

International community efforts have been demonstrated through shared tasks involving clinical notes from CDWs. In the included articles, the Informatics for Integrating Biology and the Bedside (i2b2) obesity challenge focused on obesity and its 15 most common comorbidities through a multiclass multilabel classification task [204,205]. Another i2b2 challenge held in 2009 concerned extracting medication information from clinical text [202,210]. Three tasks were proposed in the fourth i2b2 or Department of Veterans Affairs shared-task and workshop challenge: extraction of medical problems, tests, and treatments; classification of assertions made on medical problems; and classification of a relationship between a pair of concepts that appear in the same sentence where at least 1 concept is a medical problem [202]. These i2b2 shared tasks relied on deidentified discharge summaries from the Partners HealthCare research patient data repository. The 2018 National NLP Clinical Challenges (n2c2) shared-task workshop presented a cohort selection task for clinical trials [203].

Previously presented NLP tasks and methods were applied to medical data in different languages, with the majority being in English (153/194, 78.9%; Table 2).

Multimedia Appendix 3 presents the CDWs used in the publications presented in this review. Overall, the oldest CDWs, such as the Columbia University Irving Medical Center CDW, Mayo Clinic, and the Partners HealthCare research patient data repository, are the ones that reuse the most textual data and contribute the most to developing the application of NLP on EHR data.

**Table 2:** Language of the data used in the papers (n=194).

Data language	Publications, n (%)	References
English	153 (78.9)	[15-17,19-25,27-38,41-48,50,51,53-68,71,72,74,75,78-80,83-88,90-92,96,97,99-112,114,116,119-124,126-135, 137,140,142-149,151-154,156-159,161,162,165-176,179,181,184-187,189-192,194-196,198,200-208]
French	27 (13.9)	[39,49,52,73,76,77,81,89,93,94,113,115,118,125,136,138,139,155,163,164,177,178,182,183,188,193,197]
German	9 (4.6)	[18,26,69,95,117,150,160,180,199]
Korean	3 (1.5)	[40,65,82]
Japanese	1 (0.5)	[98]
Not mentioned	2 (1)	[70,141]

## Discussion

### Principal Findings

As CDWs become more prevalent and are adopted in many countries, they open up opportunities for clinical NLP to flourish. This review shows that the use of NLP on data from CDWs is primarily focused on extracting information from clinical texts and identifying patients. Depending on the task, various methods can be used, from symbolic methods to machine learning and deep learning techniques. The oldest CDWs are associated with the most numerous publications. This shows that the use of NLP is not a 1-time event but is intended to be established in the long term. It contributes to the continuous quality improvement of data made available in CDWs.

Symbolic and linguistics methods have still been widely used in recent years, despite the preponderance of deep learning approaches that have shown excellent results across a majority of tasks. This shows that some tasks can be partially achieved with classical NLP techniques, such as regular expressions and pattern matching that exploit specialized lexica such as drug lists and terminologies. Existing information extraction tools such as cTAKES, MedLEE, and MetaMap offer easy handling and satisfactory results. As a result, they are often used for processing English language clinical text.

Interestingly, the number of data languages presented in our review is quite low—only 5 languages: English, French, German, Korean, and Japanese. This can be explained by three factors: (1) CDWs are not cited as data sources in articles, resulting in a bias related to queries; (2) CDWs are operational in another country, but NLP has not yet been used on these data; and (3) CDWs have not yet been adopted in every country.

### Opportunities and Challenges

Although NLP methods are becoming increasingly popular, there remain challenges within the clinical field. This review demonstrates that the use of NLP in CDWs is becoming more frequent over time. However, CDWs still rarely provide open access for NLP research owing to medical data confidentiality. A first step to partially overcome the privacy constraints could involve working on deidentified or anonymized data from CDWs, as has been done in some recent shared tasks [202,204,205,210]. These shared tasks, crucial for making advances in medical NLP research, are too scarce, particularly for languages other than English [9]. Providing an appropriate measure to respect patient privacy should encourage collaboration among hospital and NLP research teams and facilitate access to clinical data.

The global movement is toward the structuring and interoperability of clinical data; yet, the finer points of medical reasoning are always expressed in textual reports, and such information cannot always be structured. The increase in NLP approaches applied to clinical data could lead to major advances in clinical research, both to identify the populations of interest and to retrieve relevant information of these patients for clinical research. NLP could also have a positive impact on the daily life of caregivers by speeding up access to information contained in patient EHRs using automated tools for the summarization of patient history. Indeed, caregivers invest a significant amount of time recording information gathered during care delivery in textual reports. Surprisingly, they also dedicate an equivalent amount of time sifting through numerous documents to retrieve this information when needed.

Structured or semistructured data stored in CDWs provide information about patient follow-up and can serve as a valuable resource for developing or enhancing NLP systems. Indeed, temporal data can offer guidance on where the information is most relevant in the text. In addition, other data such as PHI, including names, surnames, and addresses, can be used as a starting point in NLP systems.

Clinical data are a use case for NLP research. They possess the advantage of being accessible in multiple languages owing to the global nature of medical care. This accessibility enhances research efforts focused on multilingualism. Such data are available in abundance, facilitating the acquisition of effective clinical text representations that can be applied in deep neural networks to learn relevant concept models. Clinical data fall within the category of specialized domains or languages designed for specific purposes. They share certain properties, such as specific knowledge, uses, and discourse. This also entails undertaking specific tasks such as deidentification or anonymization.

The analysis of the literature conducted here highlights the need for further development of CDWs, with a stronger integration of NLP applications throughout the entire data value chain.

### Limitations

The NLP tasks identified in this review cover only a small part of all existing NLP tasks in the general domain. These tasks globally reflect the primary needs in clinical research, such as identifying the study population and extracting clinical information for a defined population. Other tasks, such as context analysis and language modeling, have been widely studied in the general domain NLP but are less prevalent in the clinical domain. In recent years, transformer-based approaches have emerged as the state-of-the-art methods for most NLP tasks. However, this review indicates that these methods have not fully spread to the clinical domain. This demonstrates a gap between methods that are well established in the general domain NLP and their adoption in specific domains such as the clinical domain.

This review focuses on 2 very specific subjects from different emerging domains: clinical NLP and CDWs. This combination of subjects implies the use of multiple bibliographic databases and the aggregation of multiple queries to ensure good coverage of the literature. Some bibliographic databases cover a wider range of articles and include articles already present in other more specialized sources. To avoid having a surfeit of duplicate articles, we prioritized the use of the most encompassing bibliographic databases: Google Scholar and PubMed. This introduces a bias of completeness because relevant articles could be missing from the selected bibliographic databases and be present in others we did not use in this review, such as Scopus, Web of Science, and Embase.

There is another bias of completeness related to the search by keywords in the bibliographic databases. A given concept can be expressed in various ways in natural language, using different keywords. The choice of keywords is crucial to aim at both high specificity and high sensitivity, even if the selected keywords are searched in the whole paper. In this review, we used very broad keywords to have the highest sensitivity but at the expense of specificity (n=194, 14.34% relevant articles among 1353 articles identified from the queries).

### Conclusions

CDWs are central to the secondary use of clinical texts for research purposes. Our review highlights the growing interest in computerized health data, particularly in clinical texts, where NLP is used to address various clinical tasks. These tasks include patient identification and information extraction, as well as clinical NLP tasks such as language modeling, context analysis, and EHR deidentification. The broad spectrum of NLP approaches has been effectively leveraged, ranging from symbolic methods to machine learning and deep learning methods. Despite the prevalence of pretrained language models in the broader NLP domain, symbolic and linguistics methods have continued to be used in recent years. In the realm of clinical NLP for CDWs, the trends align with global NLP patterns, where resources and methods are predominantly developed for the English language. The development of NLP in the medical field will require cooperation between health care and NLP experts.

## Appendix

Multimedia Appendix 1 PRISMA (Preferred Reporting Items for Systematic Reviews and Meta-Analyses) checklist.

Multimedia Appendix 2 Search queries used in PubMed, Google Scholar, and ACL Anthology to retrieve publications for inclusion in this systematic review.

Multimedia Appendix 3 Clinical data warehouses from which data have been used in a publication.

## Abbreviations

BERT: Bidirectional Encoder Representations from Transformers
BiLSTM-CRF: bidirectional long short-term memory–conditional random field
CDW: clinical data warehouse
CNN: convolutional neural network
CRF: conditional random field
cTAKES: clinical Text Analysis and Knowledge Extraction System
EHR: electronic health record
ELMo: embeddings from language models
GloVe: global vectors for word representation
i2b2: Informatics for Integrating Biology & the Bedside
ICD-9: International Classification of Diseases, Ninth Revision
LSTM: long short-term memory
MedLEE: Medical Language Extraction and Encoding System
n2c2: National NLP Clinical Challenges
NCBO: National Center for Biomedical Ontology
NER: named entity recognition
NLP: natural language processing
PHI: protected health information
PRISMA: Preferred Reporting Items for Systematic Reviews and Meta-Analyses
SNOMED-CT: Systematized Nomenclature of Medicine–Clinical Terms
SVM: support vector machine
TF-IDF: term frequency–inverse document frequency
UMLS: Unified Medical Language System
