# Supplementary material for: Applying Natural Language Processing to Textual Data From Clinical Data Warehouses: Systematic Review
Source: JMIR Med Inform. 2023 Dec 15;11:e42477. doi: 10.2196/42477 (PMC10757232; doi:10.2196/42477)
Supplement: Multimedia Appendix 2 [file medinform_v11i1e42477_app2.docx]

*PubMed queries*

PubMed offers advanced facilities of search compare to ACL Anthology and Google Scholar. We easily cross-referenced the two topic of interest and built the following query:

- Query: CDWs and NLP
  *(“data warehousing”[MeSH Terms] OR (“data warehouse”)) AND ((“clinical”) OR (“biomedical”) OR (“health”)))
  AND
  ((“natural language processing”) OR (“NLP”) OR (“text mining”))*

*ACL Anthology query*

For ACL Anthology, we constructed three queries. Because NLP is the bibliographic domain covered by ACL Anthology, keywords related to NLP were excluded from the queries. The results of these queries were crossed and merged to be considered as a single request:

- Query 1: *“clinical data warehouse”*
- Query 2: *“health data warehouse”*
- Query 3: *“biomedical data warehouse”*

*Google Scholar query*

For Google Scholar, we constructed three queries. For each query, we ran a similar query replacing “natural language processing” with its acronym “NLP”. The results of these queries were crossed and merged to be considered as a single request:

- Query 1: *“clinical data warehouse” “natural language processing”*
- Query 2: *“biomedical data warehouse” “natural language processing”*

Query 3: *“health data warehouse” “natural language processing”*
