# Supplementary material for: Applying Natural Language Processing to Textual Data From Clinical Data Warehouses: Systematic Review
Source: JMIR Med Inform. 2023 Dec 15;11:e42477. doi: 10.2196/42477 (PMC10757232; doi:10.2196/42477)
Supplement: Multimedia Appendix 3 [file medinform_v11i1e42477_app3.docx]

Table S1. Clinical Data Warehouses (CDWs) from which data has been used in a publication.

| Data warehouse | Paper  count | Country | Date | Cited in this paper |
| --- | --- | --- | --- | --- |
| ACORN data warehouse | 1 | Memphis, Tennessee, USA | 2004 | [130] |
| Albany Medical Center data warehouse | 1 | Albany, New York, USA | - | [121] |
| Amsterdam University Medical Center | 1 | Amsterdam, Netherlands | - | [179] |
| Antwerp University Hospital (UZA) | 2 | Edegem, Belgium | - | [24,166] |
| Assistance Publique – Hôpitaux de Paris (AP-HP) | 6 | Paris, France | 2017 | [47,50,92,93,112,114] |
| Boston Children’s Hospital data warehouse | 2 | Boston, Massachusetts, USA | - | [99,158] |
| Carolina Data Warehouse for Health | 2 | Chapel Hill, North Carolina, USA | 2009 | [64,142] |
| Centre hospitalier universitaire de Sherbrooke | 1 | Sherbrooke, Québec, Canada | - | [100] |
| Columbia University Medical Center clinical data warehouse | 30 | New York, USA | 1994 | [18,22,25-27,30-34,48,49,59,61,72,101-103,119,134,141,143,155,157,158,174,175,178,195,197] |
| Enterprise Clinical Research Data Warehouse Hannover Medical School | 1 | Hannover, Germany | 2011 | [116] |
| Entrepôt de données du CHU de Tours | 1 | Tours, France | 2019 | [87] |
| Georges Pompidou European Hospital | 4 | Paris, France | 2008 | [74,117,187,196] |
| Houston METEOR | 5 | Houston, Texas, USA | 2012 | [52,57,120,122,123] |
| Indiana Network for Patient Care | 1 | Indianapolis, Indiana, USA | 1998 | [54] |
| Intermountain Healthcare enterprise-wide data warehouse | 3 | Salt Lake City, Utah, USA | 1998 | [16,85,144] |
| Kaiser Permanente Southern California | 1 | South California, USA | - | [73] |
| Korian (private group) | 1 | France | 2010 | [75] |
| Loyola University Medical Center | 4 | Illinois, USA | 2003 | [13,19,127,133,167] |
| Mayo Clinic | 24 | Rochester, Minnesota, USA | 2005 | [35,40,42-44,66,76,83,  84,88,104,128,136,145,146,160,161,171-173,  180,183-186] |
| McGill University Health Centre | 2 | Montréal, Québec, Canada | 2019 | [95] |
| Medical University of South Carolina (MUSC) Research Data Warehouse | 3 | South Carolina, USA | 2013 | [126,153,156] |
| Mount Sinai Hospital Data Warehouse | 4 | New York City, USA | 2011 | [62,77,129,165] |
| Northwestern Enterprise Data Warehouse | 2 | Chicago, Illinois, USA | 2007 | [55,78] |
| Osaka University Medical Hospital | 1 | Osaka, Japan | - | [97] |
| Paris Necker Children’s Hospital | 7 | Paris, France | 2017 | [124,135,137,138,176,  177,181] |
| Partners Healthcare Research Patient Data Repository | 13 | Boston, Massachussetts, USA | 2002 | [20,36,53,56,148,170,  180,188,200-204] |
| Rennes University Hospital | 2 | Rennes, France | 2018 | [37,79] |
| Rouen University Hospital | 3 | Rouen, France | 2019 | [162,163,192] |
| Samsung Medical Center | 3 | Seoul, South Korea | - | [38,189,207] |
| Seoul National University Hospital (SUPREME) | 2 | Seoul, South Korea | - | [63,80] |
| Stanford Medicine Research Data Repository (STARR) | 8 | Stanford, California, USA | 2008 | [46,51,81,131,153,168,169,190] |
| STRIDE Clinical Data Warehouse | 10 | Stanford, California, USA | 2008 | [28,91,96,98,105-108,  113,173] |
| University Hospital of Erlangen (UHE) | 1 | Erlangen, Germany | 2009 | [94] |
| University Hospital of Bordeaux | 2 | Bordeaux, France | 2019 | [154,182] |
| University Hospital of Würzburg | 2 | Würzburg, Germany | - | [149,198] |
| University of Arizona, Banner University Medical Center | 1 | Arizona, USA | - | [199] |
| University of Arkansas for Medical Sciences Epic MER system | 1 | Arkansas, USA | 2019 | [41] |
| University of California, Irvine medical center clinical data warehouse | 1 | California, USA | 2011 | [23] |
| University of Florida (UF) Health Integrated Data Repository | 3 | Florida, USA | - | [12,82,205] |
| UT-Physicians | 7 | Houston, Texas, United States | - | [21,44,109,110,158,193,194] |
| UW Health, School of Medicine and Public Health of the University of Wisconsin-Madison | 1 | Madison, Wisconsin, USA | - | [58] |
| VA Corporate Data Warehouse | 9 | USA | 2006 | [17,39,89,90,125,132,  147,164,206] |
| Vanderbilt University Medical Center Synthetic Derivative | 3 | Nashville, Tennessee, United States | 2014 | [20,111] |
